# Assessing Environmental Risks for Established Invasive Weeds: Dalmatian (Linaria dalmatica) and Yellow (L. vulgaris) Toadflax in North America

**DOI:** 10.3390/ijerph8072828

**Published:** 2011-07-13

**Authors:** Sharlene E. Sing, Robert K. D. Peterson

**Affiliations:** 1 USDA Forest Service, Rocky Mountain Research Station, 1648 South 7th Avenue, MSU Campus, Bozeman, MT 59717-2780, USA; 2 Department of Land Resources and Environmental Sciences, Montana State University, 334 Leon Johnson Hall, Bozeman, MT 59717, USA; E-Mail: bpeterson@montana.edu

**Keywords:** invasive species, risk analysis, exposure assessment, ecological risk, *Linaria*

## Abstract

Environmental risk assessments characterizing potential environmental impacts of exotic weeds are more abundant and comprehensive for potential or new invaders than for widespread and well-established species such as Dalmatian (*Linaria dalmatica* [L.] Mill.) and yellow (*L. vulgaris* Mill.) toadflax. Specific effects evaluated in our assessment of environmental risks posed by yellow and Dalmatian toadflax included competitive displacement of other plant species, reservoirs of plant disease, animal and insect use, animal toxicity, human toxicity and allergenicity, erosion, and wildfire. Effect and exposure uncertainties for potential impacts of toadflax on human and ecological receptors were rated. Using publicly available information we were able to characterize ecological and human health impacts associated with toadflax, and to identify specific data gaps contributing to a high uncertainty of risk. Evidence supporting perceived negative environmental impacts of invasive toadflax was scarce.

## 1. Introduction

Risk assessment for invasive weeds has focused primarily on assessing the risk of new species invading and/or establishing in previously uncolonized locations [1–4]. Although this approach is an important predictive tool in global efforts to prevent the invasion and establishment of new weed species, it addresses only one aspect of the risks posed by exotic weeds. Another aspect is the risks posed by invasive weeds that have already become established. These weeds typically are perceived as exerting significant and largely negative economic and environmental impacts. Yet, invasive weed control programs are commonly implemented without clear ecological or economic evidence to support the need to take action [5]. Risk assessments evaluating known and potential environmental consequences of established exotic invasive weeds therefore are clearly warranted.

**Figure 1:**
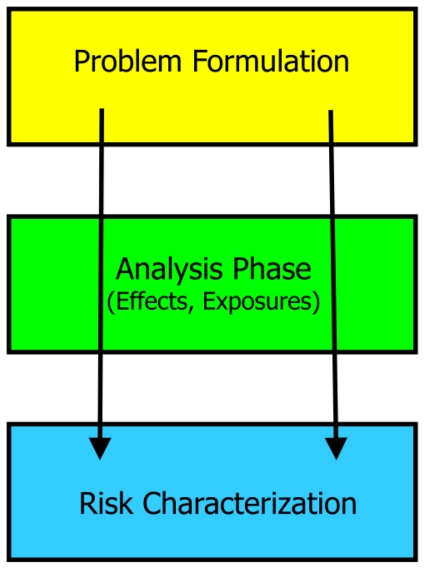
The risk assessment paradigm.

Risk assessment is a valuable framework and scientific activity from which we can measure, communicate, and make decisions about the ecological and human health impacts associated with introduced pest species [6]. However, assessing risks associated with biological organisms can be challenging due to the often unpredictable movement and reproduction of live entities. Risk assessment is a formalized basis for the objective evaluation of risk in a manner where assumptions and uncertainties are considered and clearly presented [7]. Risk assessment represents three major phases: problem formulation, data analysis (exposure and effects assessment), and risk characterization [6] (Figure 1). The problem formulation phase establishes the goals, breadth, and focus of the assessment. This phase often results in the production of a conceptual model. The data analysis phase contains the effect and exposure assessment steps. Effect assessment (also termed hazard identification) is the characterization of the inherent ability of the stressor to impact ecological receptors (entities interacting directly or indirectly with the stressor). Exposure assessment is the characterization of the interactions of the stressor with ecological receptors. The risk characterization phase is the consideration of the joint property of effect and exposure to determine risk or to determine what additional data are needed to calculate risk or refine risk estimates [7].

Although most published approaches to risk assessment of exotic pest species characterize the probability of introduction and/or establishment in new environments, substantial value can also be captured by conducting risk assessments for pest species that have already become established [8]. Consequently, we present here risk assessments focusing on ecological and human health risks associated with the invasive exotic weeds Dalmatian toadflax (*Linaria dalmatica* [L.] Mill.) and yellow toadflax (also known as common toadflax, *L. vulgaris* Mill.) (Plantaginaceae). Further, we address the utility of the risk assessment paradigm for assessing these types of risks.

## 2. Approach

### 2.1. Problem Formulation

**Figure 2:**
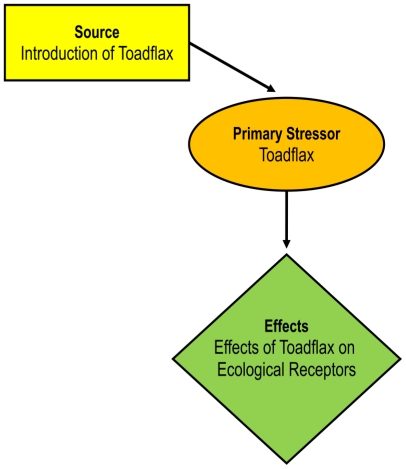
Conceptual model of risks associated with Dalmatian and yellow toadflax in North America.

An integral aspect of the problem formulation phase for any risk assessment is the development of a conceptual model (Figure 2). In this case, the source of environmental risk is the invasion and colonization of North America by non-native toadflax. Dalmatian and yellow toadflax plants are primary stressors with the potential to impact the environment through their effects. We have defined the environmental effects caused by toadflax plants as both positive and negative potential impacts on a range of ecological receptors (Figure 2). Specific effects identified were competitive displacement of other plant species, reservoirs of plant disease, animal use, animal toxicity, human toxicity and allergenicity, erosion, and wildfire.

Exposure was assessed by considering the interactions of toadflax plants (*i.e.*, the primary stressors) and ecological receptors. Specifically, we assessed the distribution and abundance of toadflax plants and how they contact or co-occur with the identified ecological receptors.

When appropriate, risks in this study were assessed by integrating effect and exposure. Where possible, this integration was done quantitatively. For certain identified risks, calculations were not possible because either exposure or effect could not be quantified. For these cases, risks were evaluated based on a weight-of-evidence, qualitative approach. In other cases, risks could not be assessed qualitatively or quantitatively because of a critical lack of data. We discuss these cases in terms of future data needs.

### 2.2. Description of the Primary Stressors

#### Species descriptions

Dalmatian toadflax, *Linaria dalmatica* (L.) Mill., is a short-lived perennial herb with a native range extending from southeastern Europe through southwestern Asia [9–11]. Dalmatian toadflax is classified as a noxious weed or weed seed in 12 U.S. states and three Canadian provinces [12–14]. American taxonomic authorities recognize two subspecies of Dalmatian toadflax that are primarily differentiated by native range: the widespread Eurasian *Linaria dalmatica* spp. *dalmatica* (L.) P. Mill., and *L. dalmatica* ssp. *macedonica* (Griseb.) D.A. Sutton, restricted to the mountains of southern Macedonia [11,14,15].

Yellow or common toadflax, *Linaria vulgaris* (L.) Mill., is also a short-lived perennial herb with a more extensive native range than Dalmatian toadflax, encompassing most of Europe and northern Asia [13,16]. *Linaria vulgaris* is readily distinguished from *L. dalmatica* by narrower leaves and smaller yellow and orange flowers [17]. Yellow toadflax is classified as a noxious weed or weed seed in 10 U.S. states and four Canadian provinces [12–14].

Formerly members of the figwort family (Scrophulariaceae), Dalmatian and yellow toadflax, along with all remaining *Linaria* congeners have been reclassified as members of the plantain family (Plantaginaceae) based on extensive molecular phylogenetic analyses [18–21].

#### Physiological range

Optimal growing conditions for Dalmatian toadflax occur in cool, semi-arid climates in dry, coarse (sandy, rocky or gravelly) soils with a neutral to slightly alkaline pH [10]. Dalmatian toadflax is typically found in open, sunny, rocky locations at altitudes ranging from near sea level to 2,800 m in uncultivated fields, vineyards, mountain meadows, ridges of sand hills and limestone mountains throughout the native range [9]. Outside of the native range, Dalmatian toadflax has adapted to a wide variety of soil types, moisture and shade conditions, and has been found under canopy covers ranging from 0–85% [22].

Yellow toadflax is thought to have originated in a steppe-type habitat characterized by dry to moderately moist sandy loam soils [17]. Yellow toadflax in North America is most commonly associated with the same substrate type occupied in the native range: dry to slightly moist, moderately to richly nutritious sandy loam soils at elevations ranging from sea level to more than 3,650 m [17,23] Whether in its native or adopted range, yellow toadflax is known as an opportunistic ruderal species that readily invades disturbed, marginal sites affected either by chronic or infrequent disturbance and offering marginal growing conditions: crop fields, roadsides, rail embankments, pastures and forest clearcuts [17]. Yellow toadflax has recently also been detected in fairly remote, undisturbed and protected habitats, invading more or less intact native plant communities in high mountain valleys, parks, and forested rangelands [23,24].

#### Geographic range

Dalmatian toadflax occurs in a latitudinal range of 33°–56° N in North America and 35°–47° N in its native range [9,11]. The native geographic range of Dalmatian toadflax extends from the Dalmatian coast of the former Yugoslavia to northern Iran. Multiple introductions to North America, either accidentally or intentionally for horticultural use, have resulted in this species’ widespread distribution across the continental United States and all Canadian provinces.

Yellow toadflax is established throughout the continental United States and in every province and territory of Canada, with a distribution extending as far north as 55°–65° N [13,17]. Yellow toadflax was historically most common in northeastern North America, with localized dense infestations occurring later in other parts of the continent. Although this weed commonly occurs throughout the Canadian Prairie provinces, it is particularly problematic in annual crops of the Peace Lowland and Aspen Park ecoregions [25].

#### North American invasion history

The widespread distribution of Dalmatian and yellow toadflax in North America today is attributed to the escape and successful establishment of ornamental specimens [11,17]. Dalmatian toadflax is believed to have been initially introduced to North America in 1894 during horticultural trials [9]. The earliest authenticated North American specimen was collected in California in 1920 [26]. Dalmatian toadflax was reportedly planted as an ornamental in Ottawa, Ontario as early as 1901 with the first confirmed Canadian herbarium specimen collected in Edmonton, Alberta in 1933 [9,27].

Anecdotal reports suggest that yellow toadflax was first introduced to North America in New England, in the 1600s [28–30]. Yellow toadflax was valued by early settlers as an ornamental and medicinal plant, and as a source of textile dye; its utility undoubtedly facilitated its spread throughout the continent as a crop seed contaminant, in baled hay, along railway corridors, and in ships’ ballasts [29,31]. The first Canadian specimen of yellow toadflax was collected in the early 1800s in southern Quebec [32]. By the early- to mid-1900s yellow toadflax had spread throughout the Prairie provinces [17].

#### Hybridization

Hybridization among *Linaria* species is fairly common [33,34]. Hybrids of *L. dalmatica* and *L. vulgaris*, *L. dalmatica* and *L. euxina* Velen. [33,35], and *L. dalmatica* (L.) Mill. and *L. gentistifolia* (L.) Mill. ssp. *genistifolia* [36] have been produced under laboratory conditions. Docherty [36] found that the hybrid progeny of self-incompatible *Linaria* species were usually fertile but also incompatible. Although naturally-occurring hybridization between *L. dalmatica* and *L. vulgaris* has not been historically recorded, field observations suggested that putative hybrid forms of *L. vulgaris* × *L. dalmatica* may be occurring in the western United States [11,37,38]. Hybridization between yellow and Dalmatian toadflax has now been confirmed from multiple field sites in Montana via molecular diagnostic techniques [39].

#### Biology/life cycle

Because Dalmatian and yellow toadflax are self-incompatible, they must be cross-pollinated by insects to produce fertile seeds [11,36,40]. Dalmatian toadflax flowers from May to October, until freezing kills the reproductive shoots; seeds are produced from late June to December [10]. Yellow toadflax flowers from mid-May to September with the seeds maturing between July and October, depending on site environmental characteristics (particularly elevation) [17]. Mature Dalmatian toadflax plants produce up to 500,000 seeds annually [10], compared to the 1,500–30,000 annual seed production of yellow toadflax plants [17]. Most yellow toadflax seeds fall within 0.5 m of the parent plant [41]. Dalmatian toadflax seeds can remain viable under room temperature dry storage and in soil under field conditions for 10 years [42].

Dalmatian toadflax seeds germinate in fall, even in the same year they were produced, or in spring [42]. Dalmatian toadflax seedlings generate flowering upright or non-flowering prostrate stems in the first growing season; prostrate stems overwinter as rosettes which then produce reproductive shoots during the next growing season [10,43]. Germination of *Linaria vulgaris* seeds typically occurs only in spring, during April and May, but can occur earlier in warm regions [17]. Viability of darker colored yellow toadflax seeds produced later in the season is generally higher than for lighter colored early season seeds [44].

Young Dalmatian toadflax seedlings have a low wilting coefficient which reduces their competitive ability against more drought-tolerant species [45]. However, older Dalmatian toadflax seedlings can monopolize moisture and nutrient resources through taproots that can grow to 50 cm within eight weeks of germination [10]. Taproot characteristics also confer significant competitive advantages to yellow toadflax. Mature yellow toadflax plants develop tap roots that can penetrate the soil to a depth of 1 m or more, along with lateral roots that extend several meters [17]. Researchers have correlated *L. vulgaris*’ well-developed taproot with species persistence and increased patch size in drought years, attributing yellow toadflax’s advantage to accessibility of deeper soil moisture reserves over shorter-rooted competing species [46].

Both species also propagate vegetatively. New Dalmatian toadflax plants arise via shoots developing from adventitious buds on lateral roots that form their own independent root systems [11]. Dalmatian toadflax is well-adapted for rapidly colonizing and dominating recently vacated niches: new shoots can arise from severed root fragments as short as 1 cm, and vegetative propagation from root buds on intact seedlings can occur as soon as two weeks after germination [10]. Yellow toadflax reproduces vegetatively from adventitious shoots produced by tap and lateral roots, root fragments and from buds in the axils of vestigial leaves at the base of floral shoots following shoot removal [47–49]. New shoots are generated from root fragments as small as 1 cm; seedlings as young as three weeks can initiate new shoots from root buds [47,49–51].

#### Phytochemistry

Tricyclic quinazoline alkaloids isolated from *Linaria* spp. include vasicine (syn. peganin), vasicinone and deoxyvasicinone [52–57]. Flavonoid glycosides and aglycons isolated from *Linaria* tissues include linarin, linarasin, acacetin, quercetin, acacetin monoglucoside, acacetin rhamnoside, and quercetin monoglucoside [58–61]. Linarin is thought to be present in the flowers but not in the leaves of *Linaria* spp. [62]. Iridoid glycosides such as antirrhinoside, isolated from the flowers and leaves of *L. vulgaris* [63], are key chemotaxonomic characters used to determine relatedness between and within the genera of Scrophulariaceae (now Plantaginaceae) [64,65]. Iridoid glycosides can be toxic or serve as feeding deterrents to generalist insect herbivores but are known to be sequestered for their own protection by specialist herbivores, including the toadflax biocontrol moth *Calophasia lunula* [66,67].

### 2.3. Effects Assessment

#### Plant displacement

Dual modes of reproduction, high seed production and long term persistence in the soil seedbank characterize invasive toadflax as opportunistic ruderal species that readily establish and persist in newly colonized sites. The extensive lateral roots of established Dalmatian toadflax plants can reach a length of nearly 3 m, providing significant stability and resistance against dislodging by biotic or abiotic forces. Toadflax grows best on disturbed soils, such as depleted rangelands, sparsely vegetated soils, roadsides, and post wildfire areas. Although both toadflax species are thought to be poor competitors in undisturbed, closed canopy areas, they are excellent competitors in open canopies. Toadflax competitively displaces other plant species primarily because the roots of mature plants so effectively capture limited soil water and moisture resources [46].

Although many overviews of Dalmatian toadflax outside of the scientific literature have discussed the species’ ability to displace desirable plant species, the scientific literature provides scant evidence of competitive displacement. Notable examples exist for yellow toadflax, which has gained status as an important crop-weed species in certain regions, such as the small grain production region in northern Alberta [68–72], and in specific crop associations, such as peppermint (*Mentha peperita*) and strawberry (*Fragaria ananassa*) [73,74].

#### Reservoirs of plant disease

Cucumber mosaic virus (CMV) is a serious pathogen of crop and ornamental plant species; weeds, including yellow toadflax, are known overwintering sites for CMV, although the potential for Dalmatian toadflax to serve as a reservoir for this and other plant diseases is not well known [75]. The disease is transmitted primarily by aphids, such as *Aphis gossypii* and *M. persicae*, and to a lesser degree via seed, cucumber beetles, parasitic plants, and mechanical contamination [76]. Vegetable hosts for CMV include cucumber (*Cucumis sativus*), tomato (*Solanum lycopersicum*), spinach (*Spinacea oleracea*), celery (*Apium graveolens*), pepper (*Capsicum* spp.), beet (*Beta vulgaris*), lettuce (*Lactuca sativa*), turnip (*Brassica rapa*), watermelon (*Citrullus lanatus*), pumpkin (*Cucurbita pepo*), broad bean (*Vicia faba*), onion (*Allium cepa*), potato (*Solanum tuberosum*), carrot (*Daucus carota sativus*), dill (*Anethum graveolens*), and parsley (*Petroselinum crispum*), while recorded ornamental hosts for CMV include chrysanthemum, delphinium, geranium, lily, marigold, morning glory, snapdragon, tulip, and zinnia.

#### Food resource and shelter—animals

Published records of livestock and wildlife species using yellow or Dalmatian toadflax as a food resource are scarce, although anecdotal reports are fairly common. Dalmatian toadflax functions as a major short-term food source for domestic goats and sheep engaged in targeted weed control grazing applications [29,77]. Cattle and horses are known to browse flowering shoots of Dalmatian toadflax [10]. Robocker [42] determined that Dalmatian toadflax seed could remain viable after transmission through the gastrointestinal tracts of cattle, thereby increasing the potential for expanded colonization and re-infestation. Although deer reportedly graze on Dalmatian toadflax shoots in fall, winter, and early spring [42], and birds and rodents are thought to feed on Dalmatian toadflax seeds [10], this weed species is not considered a favored or necessary component of any wildlife species’ diet. In addition to its occasional use as a supplemental food source, Dalmatian toadflax provides an essential source of cover, shelter and protection against predation for small animals [29].

Accounts of native insect species exploiting Dalmatian or yellow toadflax growing in North America are rare, other than for the few exotic specialist herbivores contributing to classical biological control efforts against this weed. Native insect species that did not evolve with exotic toadflax may be unable to tolerate the anti-feedant, anti-fertility and insecticidal properties of secondary metabolites such as vasicine produced by this weed [78–80]. The larval stages of one native North American species, the common buckeye butterfly (*Junonia coenia***)**, is a notable exception because it can feed on the foliage, flowers, and fruits of a variety of herbaceous species containing iridoid glycosides, including exotic (former) Scrophulariaceae species such as snapdragon (*Antirrhinum majus*) and various *Linaria* species, due to its ability to metabolize those compounds [66]. Pollinators reported at study sites in southwestern Alberta for this species included the bumble bees (*Bombus* spp.), the honey bee (*Apis millifera*), and the leaf-cutting bee (*Megachile periherta*) [81].

#### Animal toxicity

Although Polunin [82] states that toadflaxes (=*Linaria* spp.) are toxic to livestock, this assertion has yet to be experimentally substantiated. Provenza [83] suggests that neurologically mediated post-ingestive feedback is the primary determinant of food preference and consumption in ruminants; it therefore seems likely that livestock that actively avoid feeding on toadflax [43] are doing so in response to the variety of bioactive secondary compounds, including alkaloids, flavonoids, triterpenoids, steroids and iridoid glycosides, produced by these species [57,84].

Livestock ingestion of plants containing quinolinzidine alkaloids has resulted in acute poisoning in sheep, birth defects such as crooked calf disease in cattle, and prolonged recumbency [85]. Chemical analysis of *L. dalmatica* by gas chromatography has shown that it contains high levels of iridoid glycosides (as much as 17.4% dry weight) [67]. Antirrhinoside constitutes as much as 4.3% of yellow toadflax dry leaf weight [63]. Vasicine constitutes as much as 0.8% of dry leaf weight in yellow toadflax and 1.24% in Dalmatian toadflax, with alkaloid concentration varying throughout the growing season [53].

Two other quinolinzidine alkaloids frequently present in *Linaria* spp. are quercetin and acacetin [59,62]. We were unable to find any publicly available information that specified quantities of either of these compounds, or for the flavonoid linarin, in the tissues of either *L. dalmatica* or *L. vulgaris*.

#### Toxicity of crude plant material

Karimova *et al.* [86] determined that the LD_100_ in mice for a 10% infusion of *L. vulgaris* administered subcutaneously or intraperitoneally was 15 g/kg body weight (BW) in mice, and that sleep was prolonged and motor activity inhibited at a dose equivalent to 2.5–6.0 g/kg BW. In a comparatively more realistic approximation of animal consumption of toadflax under field conditions, a feeding trial performed by Zilke *et al.* [87] found no indication that yellow toadflax was toxic for mice when they were fed as much as 15% yellow toadflax in their diet.

#### Quinazoline alkaloids

Atal [88] provides a comprehensive study of the chemistry and pharmacology of the quinazoline alkaloid vasicine; subsequent study results have been assessed in updated reviews by Claeson *et al.* [89] and Rachana *et al.* [90]. Engelhorn and Püschmann [91] determined the acute toxicity of orally administered vasicine was 290 and 640 mg/kg BW in mice and rats, respectively. Atal [88] reports the LD_50_ for 25–30 g mice was 78.5 mg/kg BW i.p. (intraperitoneal) and for 120–150 g rats was 250 mg/kg BW s.c. (subcutaneous). Orally ingested vasicine, daily administered over a two week period, caused no apparent toxic effects at doses of 100 mg/kg BW(rat) and 35 mg/kg BW (6–8 kg dogs) [88]. Studies reviewed for this risk assessment assert that vasicine does not have abortifacient effects when delivered orally. We question this assertion for three reasons: (1) we have no experimental evidence characterizing either toxic or abortifacient effects of vasicine on livestock and wildlife; (2) range grazing animals will likely ingest toadflax for periods longer than the extent of the reviewed experiments (>24 days); (3) cattle conditioned to graze on toadflax do not appear to exhibit any olfactory aversion to the plants (S.E. Sing, *pers. obs*.); Burgos *et al.* [92] attribute an observed reduced body weight in treated rats to their aversion to the bitter taste of a vasicine-rich plant, *Adhatoda vasica*, added to their drinking water.

#### Flavonoids

Fernández *et al.* [93] found that the flavonoid glycoside linarin administered intraperitoneally at a dose of 14 mg/kg BW significantly depressed the central nervous system of mice, increasing the sleeping time and reducing their locomotor activity, compared with a range of other flavonoid glycosides. The quercetin dihydrate oral LD_50_ for mice is 159 mg/kg BW [94]. The acacetin intravenous LD_50_ for mice is 933 mg/kg BW [95]. Hiremath and Rao [96] report that orally administered acacetin at 50 mg/kg BW has a significant reproductive effect on rats; reduced fertility is linked to dose-dependent anti-implantation activity [95]. Flavonoid LD_50_ values have not been determined for livestock or wildlife known to graze on toadflax.

#### Iridoid glycosides

We were unable to determine effects of iridoid glucosides such as antirrhinoside on livestock and wildlife from the literature.

#### Human toxicity, allergenicity, and pharmacology

The toxicity of Dalmatian toadflax to humans has not been determined, although Pammel [97] lists yellow toadflax as an irritant. Clinical evaluations of dermal irritation or allergic reaction from contact with Dalmatian toadflax have not been reported; Hruska [98] determined that the pollen of yellow toadflax had an allergen index of 4.0 out of 10, and categorized it as a moderately allergenic species.

Yellow toadflax has been widely used in folk medicine. Continued interest in the pharmacological potential of *Linaria* species (see *Phytochemistry*, above) is reflected in numerous clinical investigations of their bioactive secondary compounds, particularly associated flavonoids. The efficacy of *L. vulgaris*-based expectorant preparations used in traditional Chinese medicine to treat coughs and asthma can be attributed to the flavonoid vasicine, a known bronchodilator [99–102]. The uterine stimulatory activity of vasicine [103,104] led to a concerted evaluation of the potential role of the compound as an abortifacient and source of fertility regulation by WHO [89]. Vasicine is also known for its expectorant, antiseptic, antiperiodic and antihelminthic properties [88,105,106].

Another flavonoid, linarin, has been investigated to determine its potential sedative and sleep-enhancing properties [93,107]. Linarin has been clinically evaluated and found to be an effective, selective acetylcholinesterase inhibitor that shows promise in the development of symptomatic medications for Alzheimer’s disease and myasthenia gravis [108].

Six classes of flavonoids are commonly consumed in human diets (vegetables, fruits, herbs and legumes) at estimated rates of 23–1,000 mg daily [109]. Toadflax-based folk medicine preparations are unlikely to be acutely toxic at the dosages traditionally administered to humans.

#### Erosion

In areas where toadflax replaces grass communities, soil erosion and surface runoff may be increased [29]. However, Dalmatian toadflax may actually decrease soil erosion on sparsely vegetated sites such as those prevalent after a wildfire event, (S.E. Sing, *pers. obs.*). To our knowledge, no studies have explicitly evaluated the interaction between toadflax and erosion.

#### Wildfire

The literature does not indicate how or if Dalmatian or yellow toadflax alters fire regimes. As with erosion, there likely are situations in which toadflax may lessen or exacerbate wildfire risk. We would extrapolate from field observations that on some steep slopes where only Dalmatian toadflax is present and because this species can remain green long into autumn, the presence of this species might be more likely correlated with a reduced rather than increased risk of fire (S.E. Sing, *pers. obs*.). Experimental results of controlled burns of individual Dalmatian toadflax plants to simulate rangeland fire conditions conducted at the United States Department of Agriculture—Forest Service Fire Effects Laboratory in Missoula, Montana suggest that it is unlikely that the type of fuel provided by this species would increase the risk or intensity of fire even in situations where the plants have fully dried down (S.E. Sing, *unpublished data*).

Jacobs and Sheley [110] found that prescribed fire increased toadflax per plant biomass and seed production, did not impact toadflax density or percent cover, but did lower the cover of perennial forbs. Treating invasive weeds with herbicide has in some cases significantly increased the probability and intensity of cheatgrass invasion and dominance, which in turn is linked to increased wildfire risk, frequency, and intensity [111]. The combined impact of broadleaf herbicide application, increased probability of cheatgrass invasion when broadleaf herbicides are used, and the reduced fire-return interval coupled with increased fire intensity associated with cheatgrass invasions could therefore severely impede the persistence or re-establishment of perennial forbs.

### 2.4. Exposure Assessment

Exposure was assessed by evaluating plausible interaction scenarios where toadflax plants as the primary stressors would come into contact with various ecological receptors. Specifically, we assessed the distribution and abundance of toadflax plants and how they come into contact or co-occur with the identified ecological receptors.

#### Plant displacement

The local density of toadflax must be evaluated to accurately assess the risk of plant displacement by toadflax for a specific area [112]. Location-specific toadflax density can then be compared to known species area or yield-loss relationships at ecologically similar but toadflax free sites to produce a first approximation of the intensity of environmental risk incurred.

Dalmatian toadflax is believed to be especially competitive with winter annuals and shallow-rooted perennials. Robocker [10] observed a 62% average decrease in dry weight biomass of grass and other forbs in toadflax infested *vs.* toadflax-free plots. In a related study, non-toadflax herbage biomass was reduced by 1.92 g (48% reduction) for each gram of toadflax/8.9 m^2^, and showed a 53% reduction in herbage biomass between plots moderately and heavily infested with Dalmatian toadflax [10]. Gates and Robocker [113] observed a range of 6–22 Dalmatian toadflax plants/0.92 m^2^ on cultivated plots seeded first with one of eight grass species: Canada bluegrass (*Poa compressa*), hard fescue (*Festuca brevipila*), orchardgrass (*Dactylis glomerata*), tall wheatgrass (*Thinopyrum ponitcum*), slender wheatgrass (*Elymus trachycaulus*), intermediate wheatgrass (*Thinopyrum intermedium*), crested wheatgrass (*Agropyron cristatum*), or beardless wheatgrass (*Elymus caninus*), then over-seeded with toadflax seeds.

**Figure 3:**
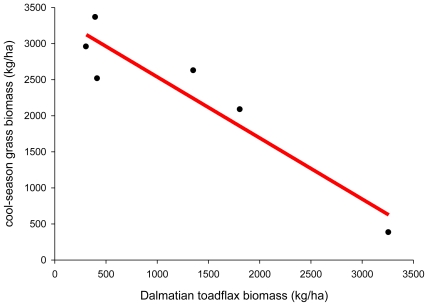
Decrease in harvested biomass of cool-season grass with increasing Dalmatian toadflax biomass. Data from Rose *et al.* [45].

Rose *et al.* [45] reported on the varying competitive abilities of five cool-season grasses: crested wheatgrass (*Agropyron cristatum*), pubescent wheatgrass (*Thinopyrum intermedium* ssp. *barbulatum*), thickspike wheatgrass (*Elymus lanceolatus*), Russian wildrye (*Psathyrostachys juncea*) and streambank wheatgrass (*Elymus lanceolatus* ssp. *psammophilus*) with Dalmatian toadflax when they were either spring- or fall-seeded on toadflax-infested plots. They determined that due to a pre-seeding application of the herbicide picloram, the mean dry weight aboveground yield on their unseeded control plots was negligible for forbs other than Dalmatian toadflax, and 380 kg/ha for grass *vs.* 3,259 kg/ha for Dalmatian toadflax, or the equivalent of 10% grass to 90% toadflax harvested biomass. All treatments showed a linear reduction in grass biomass (y = kg dry weight/ha) with increasing competitive ability of Dalmatian toadflax (x = kg dry weight/ha) (Figure 3):

(1) y=3384.5-0.8466x

**Figure 4:**
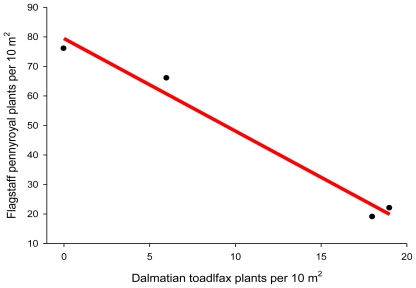
Reduction in Flagstaff pennyroyal plants (*Hedeoma diffusum* Greene) occurring for each Dalmatian toadflax plant per 10 m^2^ plot in a post-wildfire area. Data from Phillips and Crisp [114].

Phillips and Crisp [114] reported a decline in the density of Flagstaff pennyroyal plants (*Hedeoma diffusum* Greene) on study plots invaded by Dalmatian toadflax following prescribed burn treatments. Their data indicate that on toadflax-infested plots where Flagstaff pennyroyal abundance dropped below pre-burn levels, a linear reduction of 3.14 Flagstaff pennyroyal plants (y = plants/10 m^2^) occurred for each Dalmatian toadflax plant (x = plants/10 m^2^) (Figure 4):

(2) y=79.5-3.14x

**Figure 5:**
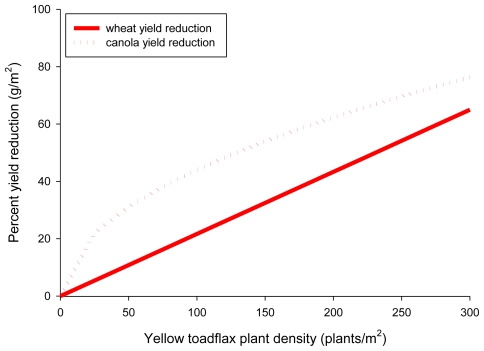
Reduction in wheat and canola harvested seed biomass with increasing yellow toadflax density. Data from O’Donovan and McClay [71] and O’Donovan and Newman [72].

The impact of yellow toadflax infestations on crop production in Alberta, Canada resulted in a 33% seed yield loss in the forage species red fescue (*Festuca rubra* L.) [70] and a 20% yield reduction in canola and wheat [71,72]. O’Donovan and McClay [71] found a linear trend with canola yield (y = g/m^2^) decreasing with increasing yellow toadflax density (x = plants/m^2^) (Figure 5):

(3) y=181.6-8.0√x

A linear relationship also described the influence of increasing yellow toadflax density (x = plants/m^2^) on decreasing wheat yield (y = g/m^2^) [72] (Figure 5):

(4) y=277-0.6x

Three studies conducted in U.S. wildland settings (Gallatin National Forest and Yellowstone National Park in Montana/Wyoming, and White River National Forest in Colorado) confirmed the ability of yellow toadflax to readily colonize and displace desired native vegetation, even in relatively intact natural habitats [23,24,115].

#### Exposure to plant disease

Cucumber mosaic virus has a broad host range; Dalmatian and yellow toadflax have broad geographic ranges. Potential host species growing adjacent to toadflax are therefore undoubtedly a commonplace occurrence. *In situ* observations of Dalmatian toadflax stems liberally infested with aphids throughout Montana suggested that a formal investigation of the weed’s potential for serving as a host for CMV was warranted. Pariera Dinkins *et al.* [116] determined through laboratory evaluations that Dalmatian toadflax is in fact susceptible to this pathogen, although this weed’s status as a reservoir for CMV has not yet been confirmed.

#### Animal exposure

Ingestion of toadflax tissue, pollen or nectar would be the most common and significant route of exposure. Species such as cattle, sheep, goats, deer, and rodents consume toadflax shoots, leaves, and flowers throughout the growing season. However, with the possible exception of goats and sheep participating in weed removal treatments, most animals do not feed primarily on toadflax [42]. Therefore, ingestion exposure would be expected to be relatively low. Similarly, insect species are typically highly host specific so that non-incidental insect exposure would be low for all but specialized herbivores.

#### Quinazoline alkaloids

Using dry-weight estimates for concentrations of vasicine in *Linaria* spp. [53], we were able to calculate values for animal grazing exposure to this compound. The average daily forage consumption by grazing wildlife and livestock is an estimate based on the amount of forage required by one animal unit (AU) for one month; the base AU value represents the amount of forage grazed by a 455 kg steer or cow in one day, equivalent to 12 kg dry forage [117]. If yellow toadflax constituted 1% of the total 12 kg daily consumed forage, then the potential daily intake of vasicine would be 0.12 kg × 0.8% (vasicine dry weight concentration in yellow toadflax), or 960 mg (2.1 mg/kg BW), respectively. Researchers at the Montana Sheep Institute determined that on three Montana farms in 2005 and 2007, Dalmatian toadflax constituted between 6 and 39% of available forage (Lisa Surber, *pers. comm.*). Using the estimated 1.24% dry weight concentration of vasicine in Dalmatian toadflax, foraging animals would consume an estimated daily dose ranging from 19.6 mg/kg BW to 127.5 mg/kg BW.

#### Flavonoids

Weathers [85] suggests that levels of stress-induced flavonoids increase in response to wilting, freezing, chewing or trampling, which are common conditions for forage plants on grazed rangeland.

#### Iridoid glycosides

Using quantitative thin layer chromatography, Nikolova-Damyanova *et al.* [118] found that the antirrhinoside content of dried, ground plant material ranged from 1.05 ± 0.05% for *L. vulgaris*. Sticher [63] determined that antirrhinoside constitutes up to 4.3% of yellow toadflax dry leaf weight, and that 50 g of dried *L. vulgaris* leaves contained 2.15 g of pure antirrhinoside. Based on the standardized animal unit consumption, animals grazing on a diet consisting of 1% yellow toadflax would ingest 11.3 mg/kg BW of antirrhinoside; a diet ranging from 10–20% yellow toadflax would deliver an estimated 113.4–226.8 mg/kg BW of the compound in the daily ration.

Jamieson and Bowers [67] report through GC and HPLC analyses that they were able to determine that combined antirrhinoside and linarioside leaf dry weight iridoid glycoside concentrations in Dalmatian toadflax ranged from 0.2–17.4%, (mean 6.28 ± 0.5 SE), with mean component concentrations of antirrhinoside at 5.02 ± 0.4 SE (max = 16.5%) and linarioside at 1.26 ± 0.1 SE (max = 6.7%). Based on the standardized animal unit consumption, animals grazing on a diet consisting of the reported 6–39% Dalmatian toadflax would ingest 79.34–516.26 mg/kg BW of antirrhinoside and 20–129.67 mg/kg BW linarioside each day. Given our increasing awareness of the occurrence of hybridization between *L. dalmatica* and *L. vulgaris* [39] it is probably appropriate to assume that iridoid glucosides levels in most North American toadflax specimens would equal or exceed those reported by Sticher [63] for yellow toadflax.

#### Human exposure

Human exposure to toadflax plants where toadflax tissue is unintentionally ingested or inhaled should be low. However, under certain circumstances, dermal contact with bioactive toadflax secondary compounds could be fairly significant, especially for organic producers or others engaged in routine or frequent, unprotected weed pulling activities. Otherwise, dermal contact with toadflax tissues such as leaves, stems, or flowers would be limited to incidental contact while cultivating crops or hiking in toadflax-infested areas. Unintentional ingestion of toadflax seed, stem, leaf, and root particles in food would be extremely low for two primary reasons: (1) only yellow toadflax has a known association with agricultural crops, and (2) produce and grain presumably would be cleaned before food from agricultural fields containing yellow toadflax is processed.

The risk of unintentional inhalation exposure to toadflax pollen seems low given that the species are insect-pollinated, not wind-pollinated [11,40]. Therefore, the pollen grains are not disseminated by the plant into the air in appreciable amounts. Intentional exposure to the flavonoids acacetin and linarin might become more prevalent in the future as these compounds show promise in the development of phytochemically-based cell cycle modulators for the treatment of various cancers in humans [119–122].

### 2.5. Risk Characterization

Despite considerable uncertainty for several effects and exposures, it is possible to qualitatively and quantitatively assess some of the environmental risks associated with toadflax. Further, although relevant dose-response data are lacking, current information suggests that toxicities to humans and animals most likely are low. More important, exposures are expected to be low, limiting contact with any toxins or allergens that may be present in toadflax. Therefore, we conclude that associated risks, too, would be low.

**Figure 6:**
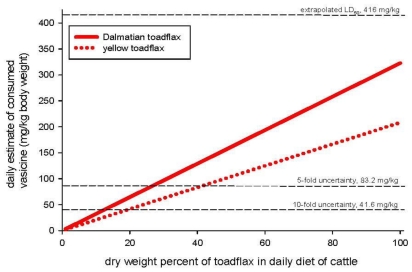
Risk characterization for estimated ingestion of vasicine by cattle delivered via *Linaria vulgaris* (0.8%) or *L. dalmatica* (1.24%) dry leaf weight concentration in standard daily food ration for grazing animals. The extrapolated LD_50_ is shown with 5- and 10-fold uncertainty levels.

Toxicity risk to animals can be conservatively estimated because there are acute oral LD_50_ values for vasicine and measured concentrations of that molecule in the plants. Using the rat LD_50_ of 640 mg/kg BW, we converted the LD_50_ to 416 mg/kg BW for cattle based on an allometric formula from Sample and Arenal [123]. We assumed that vasicine was present in yellow toadflax at 0.8% and in Dalmatian toadflax at 1.24% dry leaf weight. Therefore, assuming that grazing cattle consume 11.8 kg dry weight of forage per day, 100% of their total daily forage consumption could consist solely of yellow or Dalmatian toadflax without reaching the extrapolated LD_50_ of vasicine (Figure 6). However, if we use a 10-fold uncertainty factor so that the toxic threshold is 41.6 mg/kg BW, then cattle would only need to consume 20% yellow toadflax or 15% Dalmatian toadflax in their total daily forage diet to reach that endpoint.

The more relevant endpoints for livestock would be no-observed-effect-levels (NOELs) for acute and chronic ingestion exposures. Unfortunately, these endpoints have not been characterized for vasicine. However, Slooff *et al.* [124] suggest that 10% of the LD_50_ value can be used as a coarse approximation of the NOEL. In this case, then, the NOEL may be approximately 42 mg/kg BW and estimates of potential harm could be made from this dosage. We were unable to perform similar risk assessments for the other secondary compounds in the toadflax species because either dose-response toxicities or proportions of these compounds are not known.

Based on the effects assessment discussed above for the species’ role in erosion and wildfire risks, it is likely that the two species significantly influence these environmental risks. However, there currently is insufficient exposure and/or effect information to qualitatively or quantitatively characterize erosion and wildfire risks (or benefits).

As with many invasive weed species, competitive displacement of desirable plants is likely the most significant risk associated with both toadflax species. Although detailed competitive interaction information is lacking, we can begin to quantitatively characterize the risk of competitive displacement of plants.

Using the Flagstaff pennyroyal example discussed above, we can establish a risk threshold for Dalmatian toadflax density based on the needed protection for desirable plants. For example, if we do not want reductions in Flagstaff pennyroyal population densities to exceed 25%, we would establish a risk threshold of 6 Dalmatian toadflax plants/10 m^2^ (Figure 4). Therefore, in any scenario where both species are present, when Dalmatian toadflax plants reach a density ≥6 plants/10 m^2^, the resulting risk would be deemed unacceptable.

We can also establish a risk threshold using the biomass data on cool-season grass species and Dalmatian toadflax from Rose *et al.* [45]. For example, if we do not want biomass reductions in cool-season grasses to exceed 25% and our target biomass is 3,000 kg/ha dry weight, then we would establish a risk threshold of 1,163 kg/ha dry weight of Dalmatian toadflax (Figure 3).

In the case of yellow toadflax infestation of crops such as canola (*Brassica napus*), wheat (*Triticum aestivum*) and mint (*Mentha peperita*), the economic risk posed by yield loss seems to be the obvious impact in these agroecosystems, and therefore using conventional economic thresholds may suffice. Rather than focusing strictly on economic thresholds in these agroecosystems, however, we suggest taking a further step conceptually to consider how economic thresholds for weed density indirectly introduce a suite of environmental risks associated with weed management: nonpoint-source pollution (http://www.epa.gov/owow_keep/NPS/index.html), nontarget damage or local extinction of endemic flora [125], increased risk of desertification, *etc*. [126]. Therefore, if producers wanted to ensure that yield loss in canola or wheat was constrained at 20%, which would, for example, be a threshold that included both economic and environmental considerations, then yellow toadflax plant densities meeting or exceeding 12 and 74 stems/m^2^, respectively, would trigger a management action with its own suite of attendant direct and indirect environmental risks.

### 2.6. Uncertainties

We rated effect and exposure uncertainties for each potential impact of toadflax on human and ecological receptors (Table 1). We rated these uncertainties based on findings in the literature as well as our knowledge of environments invaded by exotic toadflax species. The effect uncertainty for competitive displacement of desirable plants is low for both toadflax species. It has been established both anecdotally and experimentally that both species are significant weeds. Although only a few studies have quantified the competitive impact of toadflax species on other plants, these studies reveal that competition can result in significant reductions in density and biomass of desirable plant species. Exposure uncertainty exceeds effect uncertainty for competitive displacement, primarily because knowledge of Dalmatian or yellow toadflax density for most locations is lacking.

Dalmatian toadflax is an acceptable host for CMV. However, considerable uncertainty remains regarding its ability to serve as a reservoir for CMV throughout its range in North America. We can conclude that uncertainty is low that yellow toadflax is a reservoir for CMV and that it is found near susceptible plant species. However, because the relationship between both Dalmatian and yellow toadflax density and disease occurrence is not known, we must consider exposure uncertainty for both to be at a medium level.

The toxicity of yellow and Dalmatian toadflax to humans and animals is poorly understood, but it is most likely low. Exposure is less uncertain. Animals generally avoid frequent or prolonged contact with non-food or non-shelter plant species, so uncertainty about exposure would be low. However, Rose *et al.* [45] suggest that Dalmatian toadflax may constitute as much as 90% of available consumable biomass for grazing cattle in heavily infested areas. Human activity patterns are well known, so exposure uncertainty is low at this time. However, if drugs are developed based on toadflax secondary compounds, human exposure and the potential to detect unanticipated deleterious interactions will increase.

The impact of both toadflax species on erosion is highly uncertain. Although Lajeunesse [29] states that soil erosion and surface runoff can be increased in situations where toadflax replaces grass communities, there are no supporting data for this position other than anecdotal information. Limited data suggest that toadflax can help stabilize sparsely vegetated areas, such as gravel pits and mines [127,128].

Currently, there are several reviews of both toadflax species in the literature, most of which have been cited in this paper. However, none has utilized the risk assessment framework to evaluate the impact of these invasive weeds on the environment. Even though few of the effects and exposures we identified currently are amenable to quantitative risk assessment approaches, we believe analysis of these invasive species within a risk assessment paradigm has considerable value.

The risk assessment framework is structured in such a way as to guide both the assessor and subsequent decision-makers through a systematic, stepwise process. This process allows for a more thorough understanding of the problem and potential solutions compared to published reviews of the pest status of these invasive species. The problem formulation, effects and exposure assessment, and risk characterization steps provide a transparent and objective understanding of the risks posed by the invasive species. More important, the paradigm, primarily through uncertainty analysis, leads to research prioritization. The uncertainty analysis has revealed several effects and exposure factors with high uncertainties (Table 1). To more fully understand post-establishment environmental risks associated with yellow and Dalmatian toadflax, it will be necessary to reduce several of these uncertainties. Therefore, if the risks currently are poorly understood, the risk assessment process itself will lead to improved understandings of risk.

For example, quantifying the concentration of biologically active phytochemicals such as linarin, quercetin, acacetin, and others previously isolated from toadflax, or characterizing the toxicity of quantified compounds such as antirrhinoside and linarioside on specific livestock and wildlife species would allow us to significantly improve our assessment of the risks posed by these weeds. At this time producers and managers are merely guessing at the potential adverse effects of toadflax consumption. In 2009, the USDA Natural Resources Conservation Services supported a Grazing Land Conservation Initiative demonstration project, “Cows Eat Weeds,” promoting methods to condition cattle to graze on Dalmatian toadflax [129]. Conversely, Davison *et al.* [130] clearly recommends against grazing cattle on toadflax.

In an initial version of Table 1 produced in 2004, there was high uncertainty associated with the ability of Dalmatian toadflax to serve as a reservoir for CMV. This led Pariera Dinkins *et al.*[116] to determine that Dalmatian toadflax can serve as a host for CMV. Consequently, this uncertainty has been reduced from high to medium. It is still uncertain whether Dalmatian toadflax is a CMV reservoir, but we now know that it can become infected by CMV. The next step would be to elucidate its status as a reservoir, and then to determine the frequency of infected plants in the environment and their proximity to susceptible, desirable plant species. Our assessments covered single compounds and did not consider potential interactive effects.

Although reducing the highly uncertain effect and exposure factors is important, some of the less uncertain factors arguably are more important to address than several of the highly uncertain factors. For example, the effect uncertainties for animal and human toxicity are much higher than the effect uncertainties for competitive plant displacement. However, more value most likely would be gained by further research on the competitive impact of both toadflax species. Although we know that both species are effective competitors in disturbed areas, the nature of this competition is still poorly understood, especially for different desirable plant species.

In addition to guiding decision-making and research on invasive weeds, a post-establishment risk assessment for a species also can be used to verify the results of introduction and establishment risk assessments for that same species. This is important because the invasiveness of an introduced species does not necessarily predict its eventual impact [131]. Consequently, post-establishment risk assessments should be important components of invasive species management databases and schemes.

Reducing or eliminating the displacement of desirable native plant species by exotic invasive species, or more broadly, “biodiversity conservation”, has been a common justification for weed control programs. However, as Sutton *et al.* [23] contend, in reference to invasive weeds in general, and yellow toadflax in particular, weed invasions commonly occur where species richness of both native and non-native species is high [132,133]. Moreover, excluding quantitative ecological risk: benefit analysis from weed management decision-making ignores the uncharacterized risks and potential secondary ecological impacts associated with specific types of weed treatment [134].

Although the scope of the risk assessment presented here is specific to the environmental risks associated with Dalmatian and yellow toadflax, risk assessments are also needed for the tactics and approaches used to manage these weed species [135]. By having risk assessments for both the invasive species and the associated management tactics, risks can be compared and more comprehensive and improved decisions can be made.

## Figures and Tables

**Table 1:** Effect and exposure uncertainty ratings for potential impacts of toadflax on human and ecological receptors.

Effect	Dalmatian Toadflax		Yellow Toadflax	

	Effect Uncertainty	Exposure Uncertainty	Effect Uncertainty	Exposure Uncertainty
Competitive Displacement	low	medium	low	medium
Reservoir of Plant Disease	medium	medium	low	medium
Animal Use	medium	medium	medium	medium
Animal Toxicity	high	medium	high	medium
Human Toxicity	high	low	high	low
Erosion	high	high	high	high
Wildfire	high	high	high	high
